# Conditionally induced RAGE expression by proximal airway epithelial cells in transgenic mice causes lung inflammation

**DOI:** 10.1186/s12931-014-0133-y

**Published:** 2014-10-29

**Authors:** B Garrett Bodine, Brock G Bennion, Emma Leatham, Felix R Jimenez, Alex J Wright, Zac R Jergensen, Connor J Erickson, Cameron M Jones, Jeff P Johnson, Steven M Knapp, Paul R Reynolds

**Affiliations:** Department of Physiology and Developmental Biology, Brigham Young University, 3054 Life Sciences Building, Provo, UT 84602 USA

**Keywords:** RAGE, Transgenic, CCSP, Lung, Inflammation

## Abstract

**Background:**

Receptors for advanced glycation end-products (RAGE) are multiligand cell-surface receptors expressed abundantly by distal pulmonary epithelium. Our lab has discovered RAGE-mediated effects in the orchestration of lung inflammation induced by tobacco smoke and environmental pollutants; however, the specific contribution of RAGE to the progression of proximal airway inflammation is still inadequately characterized.

**Methods and results:**

We generated a Tet-inducible transgenic mouse that conditionally overexpressed RAGE using the club cell (Clara) secretory protein (CCSP) promoter expressed by club (Clara) cells localized to the proximal airway. RAGE was induced for 40 days from weaning (20 days of age) until sacrifice date at 60 days. Immunohistochemistry, immunoblotting, and qPCR revealed significant RAGE up-regulation when compared to non-transgenic controls; however, H&E staining revealed no detectible morphological abnormalities and apoptosis was not enhanced during the 40 days of augmentation. Freshly procured bronchoalveolar lavage fluid (BALF) from CCSP-RAGE TG mice had significantly more total leukocytes and PMNs compared to age-matched control littermates. Furthermore, CCSP-RAGE TG mice expressed significantly more tumor necrosis factor alpha (TNF-α), interleukin 7 (IL-7), and interleukin 14 (IL-14) in whole lung homogenates compared to controls.

**Conclusions:**

These data support the concept that RAGE up-regulation specifically in lung airways may function in the progression of proximal airway inflammation.

## Introduction

Receptors for advanced glycation end-products (RAGE) propagate intracellular signaling programs following interaction with a diversity of ligands. As members of the immunoglobulin superfamily of surface pattern recognition receptors, RAGE is often considered a potent initiation factor that functions in a focal fashion. Despite acute influences, RAGE has also increasingly been implicated as a progression factor in response to the availability of advanced glycation end-products (AGEs) that accumulate during oxidant stress and when endogenous ligands including S100/calgranulins, amyloid-β-peptide, and high mobility box protein 1 (HMGB1) are augmented [1-3]. RAGE is physiologically expressed in membranes of alveolar type I epithelial cells [4] and macrophages where its signaling programs serve as an early response to perturbation. Furthermore, viscous feedback loops are common when pulmonary and non-pulmonary inflammatory lesions up-regulate RAGE signaling intermediates following stimulation [1,3,5,6].

A series of publications clearly outline discoveries that demonstrate elevated RAGE expression and signaling by pulmonary cell types when extrinsic particulates including tobacco smoke are present [7-10]. In particular, RAGE expression mediates cytokine elaboration via Ras, a GTPase that influences MAP kinase signaling intermediates that modulate the expression of pro-inflammatory NF-κB target genes [11,12]. Because RAGE and its ligands are biosynthetically up-regulated by tobacco smoke exposure, active RAGE signaling may cooperate in combined cellular responses associated with smoke-induced pulmonary inflammation. Furthermore, RAGE is significantly increased in distal lung tissue of smokers [13-15] and in the proximal airways of asthmatics that experience proximal lung inflammation [16]. It is therefore clear that a lucid understanding of the molecular aspects of RAGE signaling in the lung is critical, particularly in the sensitive upper airways of susceptible individuals.

Proximal airway inflammation and impaired airflow are inflammatory characteristics that affect 23 million Americans. Airway inflammation involves a complex interaction of cells, cytokines, chemokines and other mediators. Immune and nonimmunologic environmental factors including primary and secondhand smoke (SHS) are important triggers of proximal airway inflammation [17]. Approximately 25% to 35% of individuals with airway inflammation are current smokers [18]. It is evident that smoking or exposure to SHS increase airway sensitivity and elevate proximal airway morbidity and disease severity [17]. Prolonged exposure to tobacco smoke in patients with airway disease contributes to a decline in lung function: approximately 18% in forced expiratory volume in 1 second (FEV_1_) over 10 years [19]. Interestingly, asthmatic patients who smoke share features similar to those found in the early stages of emphysema [20]; therefore RAGE signaling observed in emphysema may also, at least in part, impact airway pathogenesis [9]. SHS from smoking parents is associated with increased airway hypersensitivity and other respiratory symptoms among school children. SHS from parents’ smoking habits also is associated with more severe disease among those children with already established asthma [21,22]. Even exposure to “light cigarette smoking” (≤10 cigarettes per day) can cause children who have airway inflammation to experience an increase of wheezing illness, especially during the first year of life, and to decreased lung function in children up to 6 years of age [23]. Because there is a clear role for RAGE in primary and SHS exposure, research into airway exacerbations by tobacco smoke should include an evaluation of RAGE biology in the proximal lung. As such, it is critical to examine how RAGE target genes influence disease presentation so that precise mechanisms that coordinate and maintain airway inflammation can be identified.

In the current study we test the hypothesis that increased RAGE expression specifically by proximal airway epithelium results in elevated inflammation. Through the utilization of a double transgenic mouse model that conditionally overexpresses RAGE in conducting airway epithelium, we demonstrate that RAGE augmentation in the absence of any additional particulate exposure leads to airway inflammation coincident with leukocyte extravasation and cytokine secretion. These data offer evidence that short-term conditional RAGE overexpression is sufficient to induce an inflammatory response; however, additional research is needed to investigate the broader applications of this model. For example, additional studies that explore a lengthened time course may demonstrate that persistent RAGE elevation in the proximal lung coordinates more robust pulmonary remodeling events. These and other studies may reveal that RAGE and its intermediates are potential targets in the treatment or prevention of chronic inflammatory airway diseases, particularly those exacerbated by tobacco smoke such as asthma, bronchiectasis, and chronic bronchitis.

## Materials and methods

### Mice

Two transgenic lines of mice were generated and mated to create conditional doxycycline (dox)-inducible mice that overexpress RAGE (CCSP-RAGE TG) [24]. One mouse line specifically included a tetracycline-inducible RAGE construct and another utilized the club cell (Clara) secretory protein (CCSP) promoter successfully used to specifically target proximal airway epithelium [25]. At post-natal (PN) day 20, mice were weaned, genotyped as similarly outlined [26], and continuously fed dox (625 mg/kg; Harlan Teklad, Madison, WI) until their sacrifice date at PN 60. Single or non-transgenic mice used as controls were also fed dox. On the day animals were sacrificed, *en bloc* lungs were inflation-fixed with 4% paraformaldehyde for histology [27], lavaged for the assessment of bronchoalveolar lavage fluid (BALF) [28], or resected prior to the isolation of total protein/RNA [29]. Mice were housed and utilized in accordance with protocols approved by the IACUC at Brigham Young University.

### RAGE quantification and histology

In order to assess whether RAGE was effectively increased in the airways of CCSP-RAGE TG mice, quantitative real time RT-PCR (qPCR) and immunoblotting were performed for RAGE using primers, antibodies, and experimental conditions already described in detail [27]. Lungs from control and CCSP-RAGE TG mice were inflation fixed, processed, and sectioned as previously outlined [30]. Slides were stained with hemotoxylin and eosin (H&E, Thermo Scientific, Pittsburg, PA) using standard techniques. Immunohistochemical localization of RAGE was performed as summarized [7]. CCSP immunohistochemistry was performed using a rabbit polyclonal IgG at a concentration of 1:500 (Seven Hills BioReagents, Cincinnati, OH) and staining for FoxJ1 was performed with a mouse monoclonal IgG at 1:500 (Seven Hills BioReagents). A TUNEL TdT-FragEL DNA Fragmentation Detection Kit (Calbiochem, Rockland, MA) was used to evaluate apoptosis wherein TUNEL positive cells were counted by blinded individuals in high power fields prior to normalization to counts observed in control animals [31].

### BALF Analysis

CCSP-RAGE TG and control mice were sacrificed and BALF was removed as described [27]. Supernatants were assayed for total protein with a bicinchoninic acid (BCA) total protein kit (Thermo Scientific). Total numbers of pelleted cells were counted with a hemocytometer and cells stained with a modified Wright-Giemsa stain (Diff-Quik; Baxter, McGaw Park, IL) were subjected to a blinded manual differential cell count in which 200 cells were counted per slide, and the percent of total cells was determined. Counting was performed in triplicate and the average was obtained.

### Quantification of pro-inflammatory cytokines

Total RNA from CCSP-RAGE TG and control lungs (n =3 per group) was isolated using the Absolutely RNA Kit (Stratagene, Santa Clara, CA) and treated with DNAse. RNA was quantified and 1 μg of each sample was converted to cDNA. Cytokines were assessed using the Mouse Inflammatory cDNA Plate Array (Signosis, Sunnyvale, CA) and the cytokine concentrations were internally normalized to 18 s RNA as outlined in the instructions. Immunoblotting for TNF-α, IL-7, and IL-14 was also performed in order to confirm differential expression in CCSP-RAGE TG mouse lungs compared to controls. Blotting was performed using antibodies for TNF-α (sc-52746, Santa Cruz Biotechnology, Santa Cruz, CA), IL-7 (sc-7921, Santa Cruz Biotechnology), and IL-14 (sc-80994 Santa Cruz Biotechnology) with 20 μg protein precisely quantified using BCA quantification and using standard methods already outlined [32]. Membranes were incubated with appropriate secondary antibodies, detected with ECL-plus (Amersham, Piscataway, NJ) and developed. Band densitometry utilized digitized images and the Un-Scan-It software package (Silk Scientific, Orem, UT).

### Statistical analysis

Values are expressed as mean ± SD obtained from at least three separate experiments in each group. Data were assessed by one- or two-way analysis of variance (ANOVA). When ANOVA indicated significant differences, the Student *t*-test was used with Bonferroni correction for multiple comparisons. Results presented are representative, and those with *p*-values <0.05 were considered significant.

## Results

### CCSP-RAGE TG mice up-regulate RAGE expression in the proximal airways

**Figure 1:**
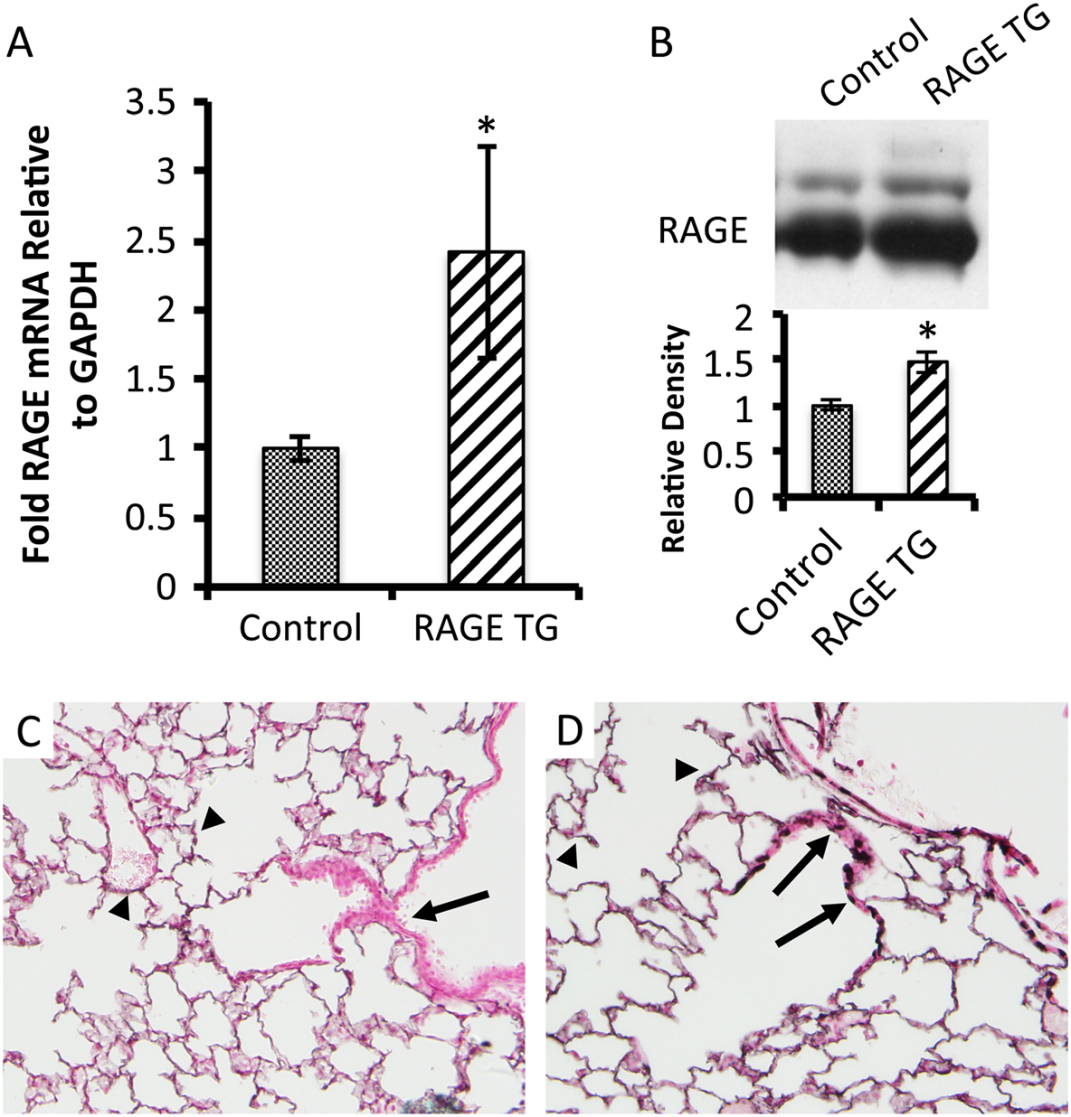
**RAGE TG mice fed doxycycline (dox) up-regulated RAGE in CCSP-expressing cells of the proximal airways.** Dox administration commenced on post natal (PN) 20 and continued until sacrifice date on PN 60. Quantitative RT-PCR **(A)** and immunoblotting **(B)** for RAGE revealed significant increases in RAGE expression following dox treatment of RAGE TG animals compared to dox-fed controls. RAGE detection using qPCR and blotting was conducted using samples with equal mRNA or protein concentrations from each animal as described. Data are representative of at least 4 animals per group and *p ≤0.05. RAGE immunohistochemistry revealed normal distal lung expression in both groups (**C** and **D**, arrowheads). No RAGE expression was detected in the airways of control mice (**C**, arrow); however, punctate RAGE expression was common in the airways of RAGE TG mice (**D**, arrows). Representative images (400× original magnification) of n =3 mice in each group are shown.

Double transgenic offspring (CCSP-RAGE TG) from CCSP-rtTA and TetO-RAGE transgenic mice were obtained and dox-mediated up-regulation of RAGE commenced on PN20, a period that coincided with the completion of lung morphogenesis. In order to assess the effectiveness of conditional RAGE up-regulation, qPCR and immunoblotting experiments were conduced using total mRNA and whole lung lysates, respectively. qPCR revealed that CCSP-RAGE TG mice fed dox from PN20 through PN60 expressed significantly more RAGE mRNA than age matched single or non-transgenic controls (Figure 1A). Lungs exposed to dox for 40 days were also evaluated by immunoblotting and the results demonstrated that RAGE protein was up-regulated when compared to control mouse lungs (Figure 1B). Immunostaining for RAGE was necessary in order to localize RAGE overexpression. Immunohistochemical staining for RAGE revealed persistent basal expression in the distal lung compartment (Figure 1C and D, arrowheads). However, punctate RAGE up-regulation by airway epithelial cells (Figure 1D, arrows) was observed in CCSP-RAGE TG mice while RAGE expression was undetectable in airway epithelial cells of control lungs (Figure 1C, arrow).

### Up-regulation of RAGE in the proximal airways did not alter lung morphology

**Figure 2:**
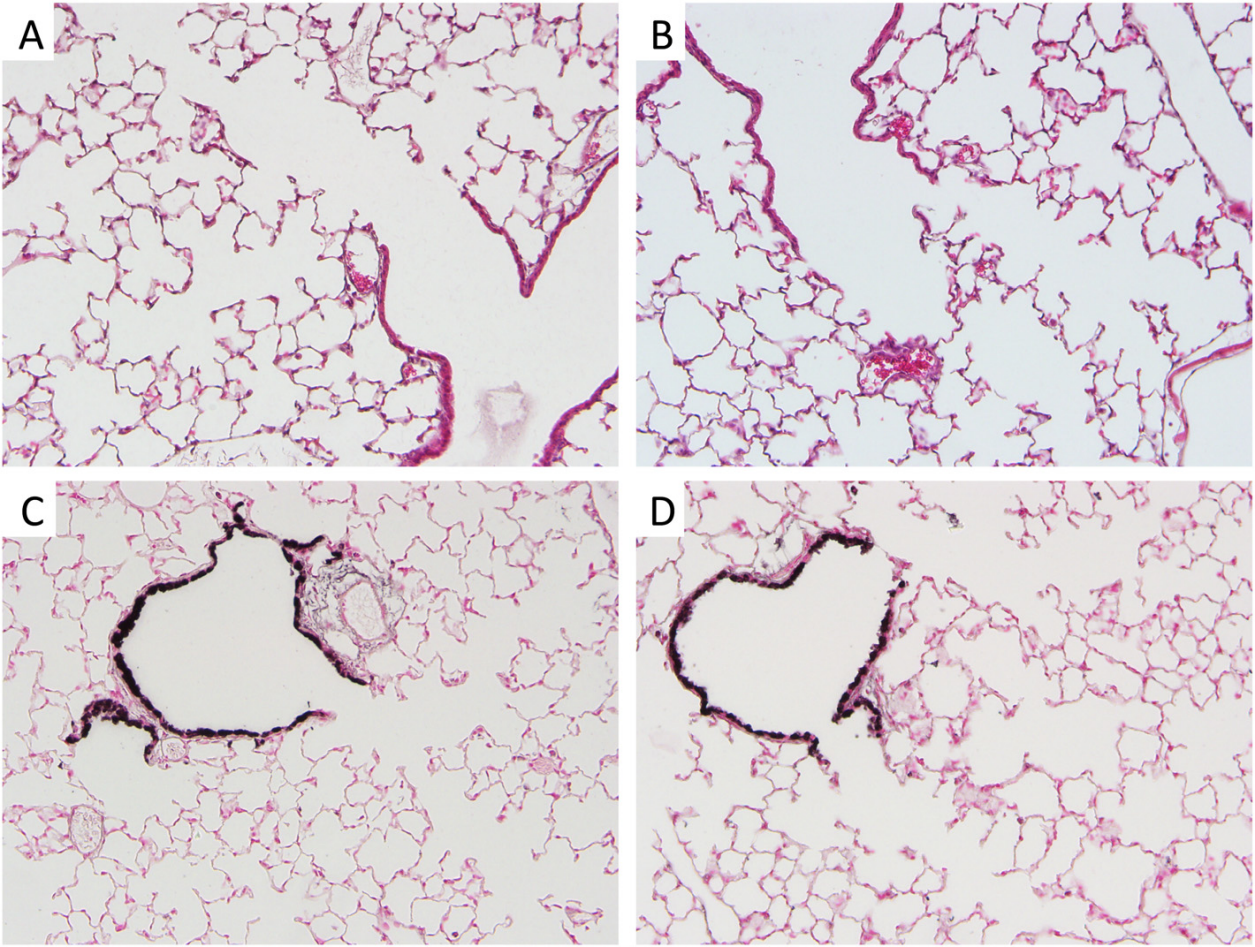
**RAGE TG mouse lungs had no significant histological alterations compared to control mouse lungs.** Control lung **(A)** and RAGE TG sections **(B)** stained with H&E revealed no morphological disturbances. Immunostaining for CCSP, a marker of club (Clara) cells in the lung airway, revealed no qualitative differences when comparing normal control lung sections **(C)** with sections obtained from RAGE TG mice **(D)**. Representative images (400× original magnification) of n = 3 mice in each group are shown.

**Figure 3:**
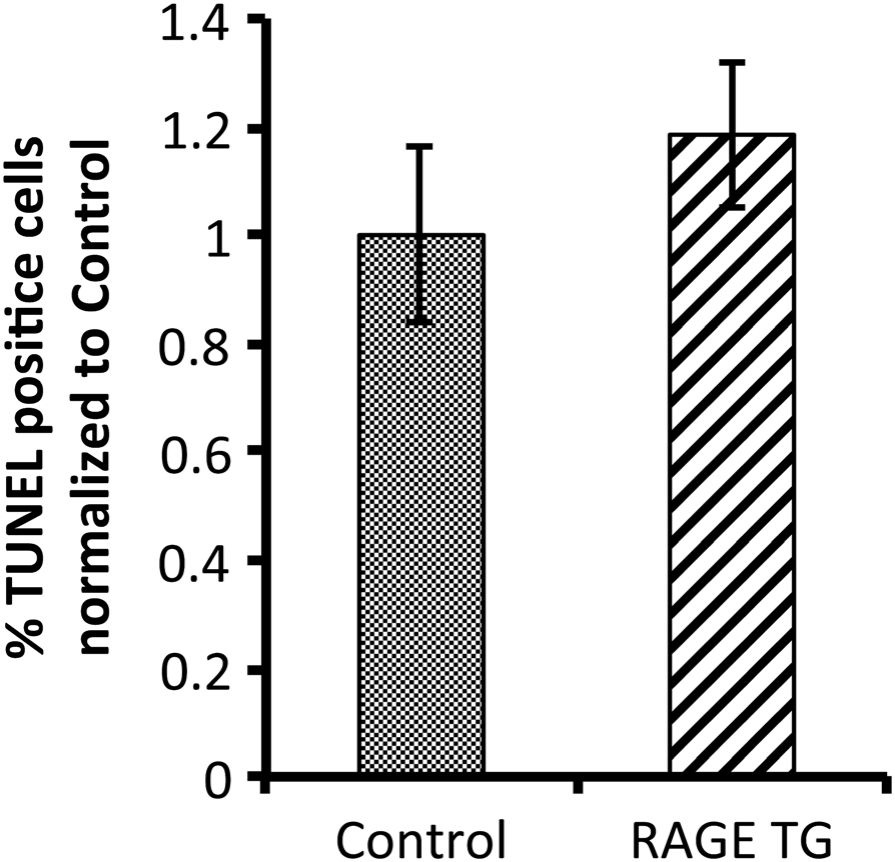
**Actively apoptosing cells uncovered by TUNEL staining demonstrated that RAGE TG mice are not significantly different from age matched control mice.** Cell counts of stained sections performed by blinded individuals demonstrated that apoptotic cells were sporadic, but no different in terms of abundance between the two groups. A minimum of three animals was evaluated in each experimental group.

Body weights of CCSP-RAGE TG animals did not significantly differ following dox administration when compared to dox-exposed control animals (not shown). Lung weights were also indistinguishable between the two groups of mice (not shown). Classic H&E staining of lung samples was completed and there were no appreciable histological differences between lungs from control animals (Figure 2A) and samples procured from CCSP-RAGE TG mice (Figure 2B) after 40 days of up-regulation. In particular, mean cord lengths and average airway wall thickness were measured [26] and these histological evaluations revealed insignificant differences in proximal airway number and appearance. Furthermore, parenchymal regions of the peripheral lung, including the alveolar compartment, were also normal in terms of size and appearance. In order to qualitatively evaluate the proximal airways, club (Clara) cells were characterized by immunostaining for CCSP. CCSP expression was not different when comparisons were made between control lung sections (Figure 2C) and CCSP-RAGE TG sections (Figure 2D). Ciliated pulmonary epithelium, the other predominant cell population in the conducting airways, was also immunohistochemically evaluated by staining for FoxJ1, a nuclear transcription factor that identifies ciliated pulmonary epithelial cells [33]. Similar to immunohistochemistry for CCSP, FoxJ1 abundance was not different when considering CCSP-RAGE TG lung sections and controls (not shown). Specialized stains for total collagen (Picro-sirius red) and proteoglycans (Periodic acid-Schiff) to identify mucus abundance revealed no differences between the groups (not shown). Even though cell-specific analyses did not suggest abnormal quantities, TUNEL staining was conduced to test whether turnover was affected. Representative staining identified sporadic apoptotic cells in lung parenchyma (not shown); however, counts revealed that despite a trend toward increased apoptosis in CCSP-RAGE TG mouse lungs, there was not a significant increase in cell death when comparing the two groups (Figure 3).

### Up-regulation of RAGE in the proximal airways induced lung inflammation

**Figure 4:**
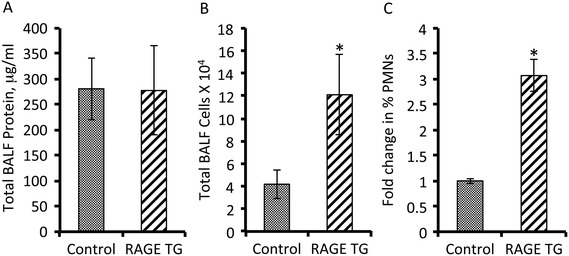
**Bronchoalveolar lavage fluid (BALF) analysis revealed increased cellularity and percent PMNs in RAGE TG compared to control mice.** RAGE TG mice did not have significant differences in total BALF protein when compared to controls **(A)**. However, the total number of leukocytes detected in BALF was statistically increased in samples from RAGE TG mice compared to controls **(B)**. A closer inspection of total cells in BALF revealed that the percentage of PMNs was significantly elevated in RAGE TG mice **(C)**. N = 6 animals per group, *p ≤ 0.05.

**Figure 5:**
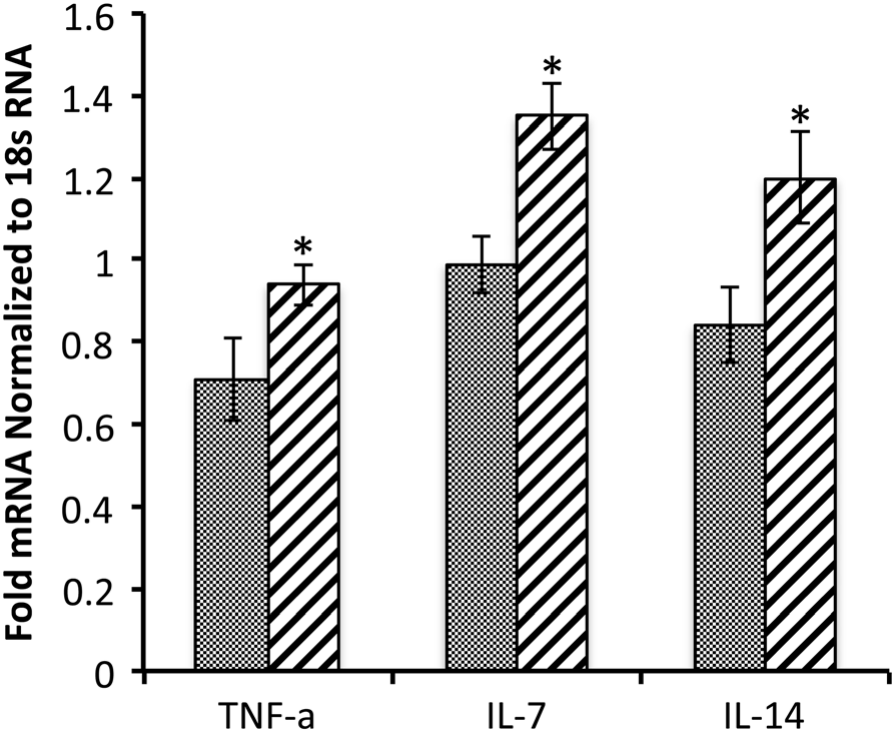
**Messenger RNA levels of the pro-inflammatory mediators TNF-α, IL-7, and IL-14 were each up-regulated in RAGE TG mouse lung lysates compared to controls.** Measurements for cytokines were standardized to 18 s RNA. A minimum of three animals were evaluated in each experimental group and *p ≤0.05.

**Figure 6:**
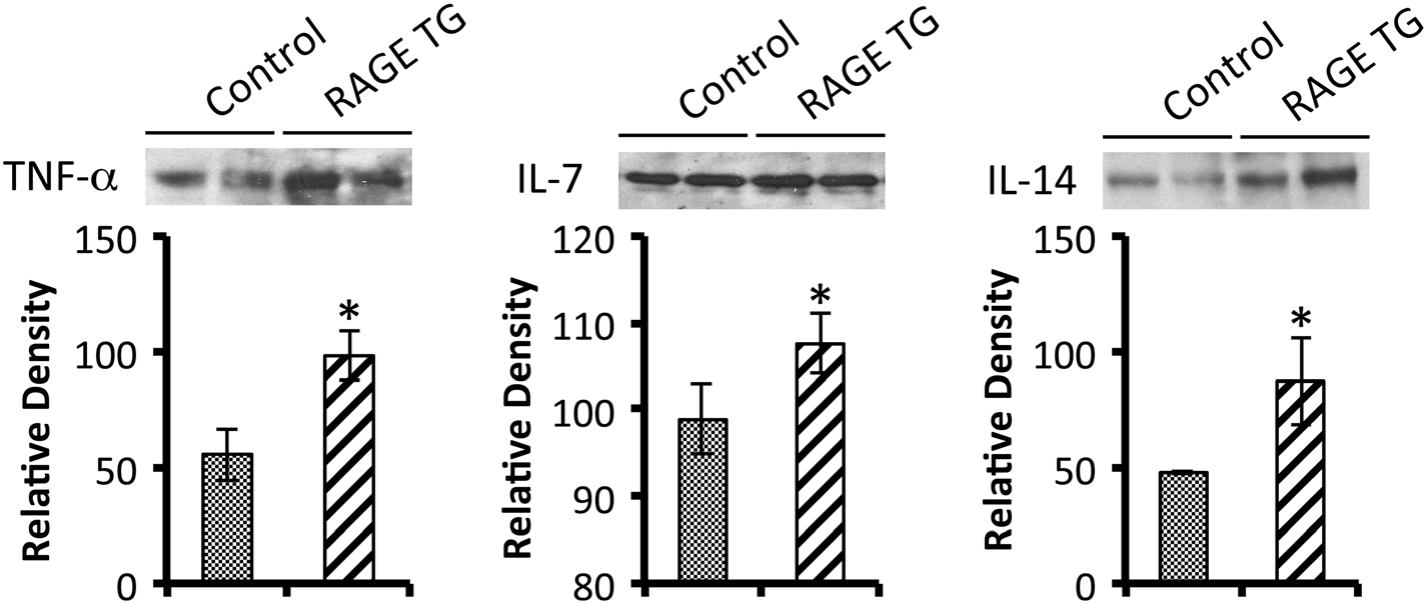
**Immunoblotting revealed that RAGE TG mice had significantly more TNF-α, IL-7, and IL-14 in total lung lysates when compared to controls.** Total protein concentrations were equal in all lanes and band densities were quantified by densitometry as outlined in the Methods section. Results are representative of n =3 mice per group, *p ≤0.05.

There was not a net increase in total protein abundance in BALF samples following 40 days of RAGE up-regulation in the proximal lung (Figure 4A). In order to assess the potential for leukocyte extravasation, total cell quantities in BALF samples were obtained prior to their morphological identification. The total number of cells in BALF from RAGE TG mice was significantly elevated when compared to controls (Figure 4B). When cells were anatomically identified, the percentage of polymorphonucleocytes (PMNs) was significantly elevated (Figure 4C), suggesting diapedesis of these cells is mediated, at least in part, by RAGE signaling. Eosinophils also trended higher in RAGE TG mice compared to controls; however, the average increase was just beyond significance (p =0.06). Altered leukocyte quantities provided the rationale for the subsequent evaluation of inflammatory cytokines implicated as participants in leukocyte chemoattraction. Characterization of mRNA isolated from mouse lung samples revealed that RAGE TG mice had significantly increased levels of TNF-α, IL-7, and IL-14 compared to control animals (Figure 5). We also discovered marginally increased expression of important Th2 related cytokines including IL-4, IL-6, and IL-13; however, such elevations in expression were not significant. In order to correlate mRNA expression with protein levels, immunoblotting for TNF-α, IL-7, and IL-14 was also completed. Compared to lung lysates from control animals, TNF-α, IL-7, and IL-14 were each up-regulated in lungs from CCSP-RAGE TG mice (Figure 6).

## Discussion

The present investigation explores the basis of RAGE function in the proximal airways and demonstrates the manifestation of inflammatory characteristics with persistent RAGE availability. The plausibility that RAGE participates in airway inflammation is only a recent development; however, research from multiple independent laboratories has demonstrated that RAGE expression increases in the airways of sensitized, inflamed lungs. For example, research by Ullah et al. reveled that RAGE and HMGB1 were both augmented in the allergic airway and that the activation of a RAGE-HMGB1 signaling axis in response to various allergens mediated allergic airway sensitization [16]. Additional research that employed blocking antibodies against HMGB1 led to the discovery that airway inflammation was ameliorated in ovalbumin (OVA)-immunized mice with hypersensitive airways [34]. Specifically, HMGB1 abrogation led to significantly less inflammatory cell abundance, mucus secretion, and collagen deposition characteristic of asthmatic lung remodeling [34]. Milutoinovic et al. also recently demonstrated that the inflammatory profiles in RAGE null mice were lessened following house dust mite and OVA-induced asthma pathogenesis [35]. These experimental outcomes support the theme that RAGE signaling inhibition may provide a promising therapeutic strategy in the alleviation of proximal airway inflammatory diseases.

Increased leukocyte abundance in the airways of RAGE TG mice was observed despite no abnormal lung remodeling compared to controls. These observations were consistent with human studies that involved the characterization of induced sputum from normal healthy individuals compared to patients with airway inflammation [36]. In particular, patients identified by higher Asthma Control Questionnaire (ACQ) results expressed significantly higher neutrophil numbers that were associated with elevated HMGB1 and RAGE expression [36]. Interestingly, a newly developed viral-induced mouse model of airway inflammation revealed a similar neutrophilic inflammatory profile in BALF assessments coincident with no abnormal lung histology [37]. While our research identifies that a short-term period of RAGE up-regulation was sufficient to induce airway inflammation, chronic studies using this model should be designed to test whether lung remodeling observed in prolonged inflammatory conditions is induced. For instance, a more chronic assessment may lead to significant increases in eosinophil counts that, with higher PMN numbers, cause histopathological remodeling of the airway. Moreover, phenotypic characterization of other leukocytes including lymphocytes and macrophages would prove insightful when considering causes of RAGE-mediated airway inflammation.

While we did not detect a significant increase in Th2 cytokines after 40 days, we observed elevated expression of TNF-α, IL-7, and IL-14 in RAGE TG mouse lungs compared to controls. TNF-α is the prototypic ligand of the TNF superfamily [38]. It is a pleiotropic molecule that centrally functions in inflammation, immune system development, apoptosis, and lipid metabolism [39]. In addition to inflammatory lung functions, TNF-α is also involved in a number of severe pathological conditions including Crohn’s disease, rheumatoid arthritis, neuropathic pain, obesity, type 2 diabetes, septic shock, autoimmunity, and cancer [40]. IL-7 was originally discovered as a growth factor produced by stromal cells that aided in the proliferation of precursor B-lymphocytes [41]. In addition to being produced by bone marrow stromal cells, IL-7 mRNA has also been detected in spleen, thymus, kidney, and epithelial cells [42]. Functionally, IL-7 has been shown to have pleiotropic effects on a variety of cell types, including cells of the B-, T-, NK-, and myeloid lineages [40]. Although less studied, IL-14 primarily enhances immune cell proliferation and it is thought to partner with TNF-α during inflammatory signaling [43]. It remains possible that Th2 related cytokines associated with eosinophilic inflammation are increased with more prolonged RAGE over-expression in the proximal lung. Accordingly, the activation of other T cell responses such as those associated with Th1, T reg, and Th17 should be considered. These responses may contribute to the refining of common Th2 responses plausibly controlling Th2-mediated eosinophil abundance trending higher after just 40 days of RAGE up-regulation. A more thorough inspection of these responses, together with Th17 modulators such as IL-17A, IL-17 F, IL17AF, IL-21, and IL-22 should be undertaken in future analyses of long term RAGE over-expressing mice.

Our discoveries related to increased TNF-α, IL-7 and IL-14 expression in RAGE TG mice supports previous research in the areas of airway inflammation disease diagnosis and progression. Thomas et al. demonstrated that TNF-α exacerbates airway sensitivity by controlling neutrophilia and it cooperates with IL-14 in increasing airway responsiveness [43]. Complimentary studies revealed that IL-7 was increased by airway epithelial cells following exposure to environmental particulates with diameters that are less 2.5 μm known to promote asthma [44]. Furthermore, IL-7 centrally functioned in the recruitment of eosinophils in chronic airway inflammatory events [45]. A link clearly exists between the current RAGE TG mice and previously published studies that thematically describe inflammatory programs involving TNF-α, IL-7 and IL-14 [43,46].

In conclusion, the present study revealed that conditional genetic up-regulation of RAGE in the proximal airways leads to the induction of an inflammatory response. RAGE up-regulation for 40 days caused expanded extravasation of leukocytes and elevated expression of cytokines implicated in inflammatory pathogenesis. The data revealed that a short period of RAGE expression was sufficient to initiate inflammation; however, further research defining cellular mechanisms that function during chronic RAGE up-regulation may aid in clarifying a more accurate model of airway inflammatory disease. Further elucidation of the sufficiency of RAGE signaling in the airways may lead to strategies for attenuating proximal airway inflammatory diseases.
